# Twin CHCH Proteins, CHCHD2, and CHCHD10: Key Molecules of Parkinson’s Disease, Amyotrophic Lateral Sclerosis, and Frontotemporal Dementia

**DOI:** 10.3390/ijms20040908

**Published:** 2019-02-20

**Authors:** Yuzuru Imai, Hongrui Meng, Kahori Shiba-Fukushima, Nobutaka Hattori

**Affiliations:** 1 Department of Research for Parkinson’s Disease, Juntendo University Graduate School of Medicine, Tokyo 113-8421, Japan; 2 Department of Treatment and Research in Multiple Sclerosis and Neuro-intractable Disease, Juntendo University Graduate School of Medicine, Tokyo 113-8421, Japan; 3 Department of Neurology, Juntendo University Graduate School of Medicine, Tokyo 113-8421, Japan; hmon@juntendo.ac.jp; 4 Department of Neurodegenerative and Demented Disorders, Juntendo University Graduate School of Medicine, Tokyo 113-8421, Japan; kshiba@juntendo.ac.jp

**Keywords:** coiled-coil-helix-coiled-coil-helix domain, OXPHOS, respiratory complexes, mitochondria, CHCH domain, MICOS, neurodegenerative diseases

## Abstract

Mutations of *coiled-coil-helix-coiled-coil-helix domain containing 2* (*CHCHD2*) and *10* (*CHCHD10*) have been found to be linked to Parkinson’s disease (PD), amyotrophic lateral sclerosis (ALS), and/or frontotemporal lobe dementia (FTD). CHCHD2 and CHCHD10 proteins, which are homologous proteins with 54% identity in amino acid sequence, belong to the mitochondrial coiled-coil-helix-coiled-coil-helix (CHCH) domain protein family. A series of studies reveals that these twin proteins form a multimodal complex, producing a variety of pathophysiology by the disease-causing variants of these proteins. In this review, we summarize the present knowledge about the physiological and pathological roles of twin proteins, CHCHD2 and CHCHD10, in neurodegenerative diseases.

## 1. Introduction

A mitochondrion is an essential organelle that converts nutrients into chemical energy by a oxidative phosphorylation (OXPHOS) reaction to provide ATP as energy in eukaryotic cells. Mitochondrion also plays multifaceted roles including cellular stress/immune response, metal ion metabolism, calcium signaling/buffering, apoptosis, and cytosolic protein degradation [1,2]. Mitochondria contain their own circular genome (~16,600 base pairs), where only 37 genes (13 OXPHOS-related proteins, 2 ribosomal RNAs and 22 tRNAs) are coded in eukaryotes [3]. Aside from the 13 OXPHOS proteins coded on the mitochondrial genome, approximately 1500 mitochondrial proteins coded in the nuclear genome are produced on the cytosolic ribosomes as precursor proteins and are imported into mitochondria via the mitochondrial import machinery [4]. 

Mitochondrial dysfunction is a hallmark of aging and a variety of disorders including ischemia/reperfusion injury in stroke and myocardial infarction, metabolic syndromes, and neurodegeneration [2,5]. Mitochondrial dysfunction is often caused by acute or chronic oxidative stress derived from reactive oxygen species (ROS) during OXPHOS reaction. Cells are equipped with a variety of stress signaling to cope with this issue. However, the failure of OXPHOS components and dysfunction of signaling against oxidative stress would lead to chronic oxidative stress, which imperils the health of organisms.

**Figure 1:**
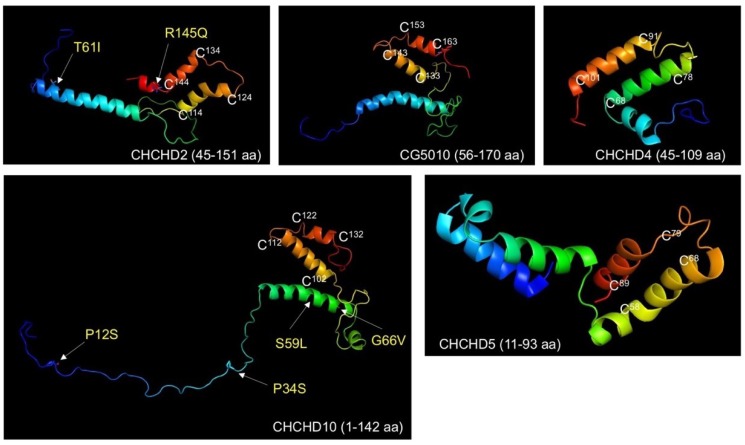
Structures of proteins containing twin CX_9_C motifs. The structures of human CHCHD4/Mia40 and human CHCHD5 were drawn from 2K3J and 2LQL deposited in the protein data bank. The predicted structures of human CHCHD2, human CHCHD10, and *Drosophila* CG5010 (an orthologue of CHCHD2/10) were depicted using RaptorX structure prediction server. The cold to warm colors indicate N-terminal to C-terminal region. The cysteine residues predicted to form disulfide bonds in twin CX_9_C motifs (white) and key residues associated with pathogenesis (yellow) are also shown. The central α-helices (see also Figure 2) and twin CX_9_C motifs are highly conserved among CHCHD2, CHCHD10, and CG5010, while there are several predicted disorder regions in these proteins, which makes it difficult to draw accurate structures. Note that the predicted structures of CHCHD2, CHCHD10, and CG5010 may not reflect the physiological conformation due to in silico analysis.

Mitochondrial proteins are generally small, which is attributed to the property of mitochondrial import machineries. Members of nucleus-encoded mitochondrial small proteins containing twin CX_9_C motifs ((CX_9_C)_2_) are imported to the mitochondrial intermembrane space (IMS) and are folded by the disulfide relay-dependent Mia40 machinery [6]. The twin CX_9_C motifs form the coiled-coil-helix-coiled-coil-helix (CHCH) domain, which is characterized by four cysteine residues, forming two disulfide bonds, stabilizing the helix-turn-helix fold (Figure 1).

Recently, mutations of the genes for mitochondrial twin CX_9_C proteins, *CHCHD2* and *CHCHD10*, were identified as being linked to the pathogenesis of Parkinson’s diseases (PD) [7], amyotrophic lateral sclerosis (ALS) and frontotemporal lobe dementia (FTD) [8]. Subsequent studies have demonstrated that *CHCHD2* and *CHCHD10* are associated with a variety of dominantly inherited neurodegenerative diseases as well as sporadic neurodegenerative diseases [9]. In this review, we summarize the physiological functions of *CHCHD2* and *CHCHD10* and discuss their pathological roles in neurodegenerative disorders.

## 2. Genetic and Pathological Overlaps between ALS and FTD, but not between ALS–FTD and PD

ALS and FTD are clinically distinct adult-onset neurodegenerative diseases. ALS patients progressively exhibit muscle weakness, wasting and spasticity caused by degeneration of the motor neurons in the motor cortex, brainstem, and spinal cord, and eventually die from respiratory failure caused by the loss of the motor neurons innervating the respiratory muscles. In contrast, FTD patients display progressive disturbances to behavior, personality, language, cognitive functions, and motor function that are associated with degeneration of the neural network in the frontal and temporal lobes of the brain. In spite of the different clinical symptoms, 15% of FTD cases also exhibit ALS phenotypes, whereas 15% of ALS cases have FTD with TAR DNA-binding protein 43 (TDP-43)-positive inclusions in the cortical neurons, and at least 50% of ALS cases show subtler cognitive and/or behavioral problems [10,11,12]. There are significant genetic overlaps between ALS and FTD, which is represented by *C9orf72* expansions, mutations of *TBK1*, *VCP*, *TDP-43,* and *CHCHD10*. Thus, ALS and FTD are thought to represent a continuous disease spectrum. Although *TDP-43* mutations are rare in ALS and FTD (<1%), neuronal inclusions of TDP-43 in affected brain regions and motor neurons are reported in the majority of ALS (up to 97%) and FTD (up to 50%) cases [13,14,15].

PD is the most common neurodegenerative movement disorder, which is characterized by rigidity, tremor, and bradykinesia—phenotypes associated with the loss of dopaminergic neurons in the substantia nigra of the midbrain. Pathologically, α-Synuclein-positive neuronal inclusions called Lewy bodies are found in the residual DA neurons. Although most PD cases are sporadic, 5–10% of patients carry monogenetic mutations. Over 20 genes or genetic loci for familial PD have been isolated, which includes *SNCA/α-Synuclein*, *Parkin*, *PINK1*, *DJ-1*, *ATP13A2*, *DNAJC6*, *EIF4G1*, *FBXO7*, *LRRK2*, *VPS35*, *PLA2G6*, *SYNJ1*, *VPS13C*, and *CHCHD2* [16,17,18]. However, unlike in the case of ALS and FTD, there are minimal or no generic or pathological overlaps between PD and ALS–FTD [19].

## 3. *CHCHD2* Mutations Linked to PD

**Figure 2:**
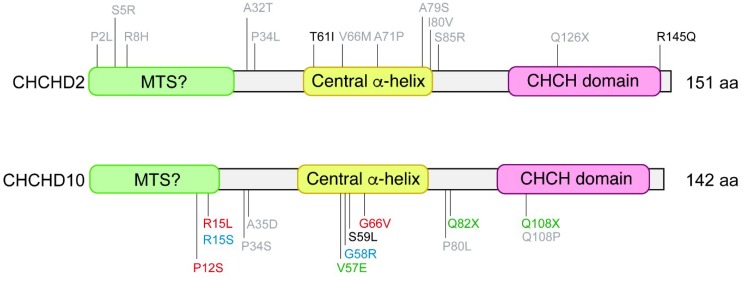
Representative disease-associated variants in the coding regions of *CHCHD2* and *CHCHD10.* (**Upper**) Coding variants in PD patients are in black [7,30,31]. Potential risk variants found in PD [7,17,30,32,33,34,35,36,37], DLB [32], AD [38,39], FTD [39], and MSA [40] patients without family history or sporadic cases are in gray. CHCHD2 T61I has been found in multiple families and has the greatest evidence for pathogenicity while R145Q has only been reported in the first case from familial PD population. Note that the pathogenicity of other variants is not fully determined due to the absence of segregation data in families and burden analysis of rare variants. (**Lower**) Coding variants in FTD patients [23,25] are in green, in patients with ALS [21,23,24], CMT2 [26,27], or SMAJ [28] in red, in ALS–FTD patients [8,22] and patients with motor neuron disease [21] in black, and in mitochondrial myopathy patients [29] in blue. Potential risk variants found in ALS [41,42,43,44,45], FTD [44], FTD with PD pathology [25], AD [46], and ALS–FTD [22] patients are in grey. Note that R15S and G58R were found in cis and there is the possibility that one or the other but not both are pathogenic [29].

*CHCHD2* mutations have originally been identified with dominantly inherited PD [7]. Recent findings demonstrate that *CHCHD2* mutations are associated with late-onset autosomal dominant PD, as well as sporadic PD and dementia with Lewy bodies (DLB). So far, two missense mutations (T61I and R145Q) and a splice-site mutation (c.300 + 5G > A) to induce non-sense-mediated mRNA decay have been identified as pathogenic variants linked to autosomal dominant PD. However, *CHCHD2* mutations identified in PD patients in Asian populations appear to be absent in Caucasians. A recent study identified three novel putative pathogenic variants (A32T, P34L, and I80V) from four western European familial PD patients [20]. Representative pathogenic and risk variants are summarized in Figure 2.

## 4. *CHCHD10* Mutations Linked to ALS-FTD

The ALS–FTD-associated S59L mutation of *CHCHD10* was first identified in a French family [8]. The patients were affected by motor neuron disease, frontotemporal dementia-like cognitive decline, cerebellar ataxia, and myopathy. Using a whole exome sequencing approach, the S59L mutation was shown to segregate with affected individuals. The same mutation was also identified in a patient of Spanish descent who presented signs of both ALS and FTD, in addition to having a family history of ALS. Therefore, *CHCHD10* has been recognized as a new ALS–FTD-causative gene. Subsequent studies have identified *CHCHD10* mutations in European populations in association with a variety of phenotypes, mostly ALS and FTD [21,22,23,24,25], but also Charcot–Marie–Tooth disease type 2 (CMT2) [26,27], spinal motor neuronopathy [28], motor neuron disease [21], and mitochondrial myopathy patients [29]. Representative pathogenic and risk variants are summarized in Figure 2.

## 5. Twin Proteins, CHCHD2, and CHCHD10 Regulate OXPHOS

**Figure 3:**
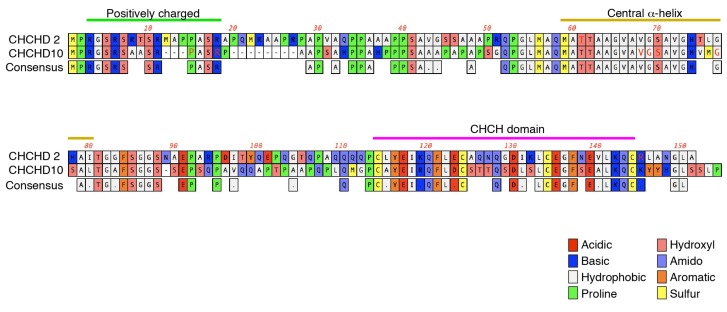
Alignment of human CHCHD2 and CHCHD10. N-terminal positively charged regions proposed as MTS, central α-helices, and CHCH domains are shown. Sequence alignment was performed by ClustalW program while α-helices and CHCH domains were predicted by the RaptorX program. Red letters in sequences indicate affected amino acids in neurodegenerative diseases.

Human CHCHD2 and CHCHD10 proteins are very similar at amino acid sequence levels (54% identity by ClustalW alignment) (Figure 3). Both proteins are highly conserved from yeast to humans [47]. In yeast, loss of *Mix17/Mic17* (orthologue of human *CHCHD2* and *CHCHD10*) results in mild reduction of cytochrome c reductase (complex III) activity and increased cytochrome c oxidase (complex IV) activity while oxygen consumption is markedly suppressed [47]. However, complex III subunits (Cor2, Cyt1, and Rip1) and complex IV subunit (COX2) levels are not changed, suggesting that Mix17 regulates the enzymatic activities of complex III and complex IV [47]. In human cultured cells, the silencing of *CHCHD2* reduces the cytochrome c oxidase activity, the oxygen consumption, and the expression of both mitochondrial DNA-encoded COX2 and nucleus-encoded NDUFB8, a subunit of complex I [48], while the NADH:ubiquinone oxidoreductase (complex I) activity is not altered. An earlier study using transcriptional association analysis in human tissues reported that genes showing correlated expression with *CHCHD10* include the respiratory complex subunits [49]. Silencing of *CHCHD10* in HeLa cells shows reduced ATP production and complex IV activity [49]. The observations of reduced oxygen consumption using yeast and human cells again suggest that the function of complex IV is mainly compromised by the suppression of CHCHD2 and CHCHD10, affecting the activity of mitochondrial protein transport and of protein expression from the mitochondrial genome.

*Drosophila* has a single copy of the *CHCHD2/CHCHD10* orthologue, *CG5010*. Loss of *CG5010* results in reduced oxygen consumption, reduced ATP production, mitochondrial cristae dilation, higher ROS production, and apoptotic degeneration in muscles. These mitochondrial phenotypes are rescued by complemented expression of human wild-type CHCHD2, but not PD-associated T61I and R145Q mutants [50].

## 6. Submitochondrial Localization of Twin Proteins

A recent study indicated that CHCHD10 is imported to the IMS by Mia40 machinery through the CHCH domain of CHCHD10, rather than the proposed MTS (1–16 aa) [45], while the requirement of both the MTS and CHCH domain is reported in other studies [51,52]. The requirement of the CHCH domain for mitochondrial import is also reported in CHCHD3, but N-terminal myristoylation is another essential modification for its mitochondrial localization [53]. Although the four cysteines of the CHCH domain of CHCHD3 are important for binding to Mitofilin in the inner mitochondrial membrane, and the sorting and assembly machinery SAM50 for correct folding on the outer mitochondrial membrane, they are not essential for mitochondrial import.

CHCHD10 is enriched at the cristae junction in the IMS and maintains the cristae organization [8]. It is a proposed component of the mitochondrial contact site and cristae organizing system (MICOS) complex, which contains MIC60/Mitofilin, MIC27/APOOL, MIC25/CHCHD6, MIC19/CHCHD3, and MIC10/MINOS1, and maintains the cristae junction [54,55], although the finding that CHCHD10 is a MICOS subunit has failed to be supported by others [51,56,57]. The immunoelectron microscopic analysis suggests that CHCHD2 and CHCHD10 are not colocalized with CHCHD3, a component of the MICOS complex [57]. Consistent with the observations, we failed to detect the direct interaction between CHCHD2 and Mitofilin or CHCHD3 [57,58]. However, CHCHD2 and CHCHD10 could indirectly maintain MICOS integrity [58].

## 7. Expressional Regulation and Tissue Expression of Twin Proteins

A transcription factor, Nurr1, is essential for the development and survival of ventral midbrain dopaminergic neurons [59,60] and activates the expression of CHCHD2, along with several complex IV subunits and mitochondrial ribosomal proteins in dopaminergic neurons [61]. Reprograming-associated transcription factors, OCT4 and SOX2, recognize the *CHCHD2* promoter, leading to transcriptional upregulation [62]. In this context, CHCHD2 expression correlates with the differentiation potential to neuroectodermal lineages in human-induced pluripotent stem cells [62]. 

In coexpression network analysis using human B cells, *CHCHD2* was predicted to be closely connected to transcription and glycolysis metabolism [63]. The roles of CHCHD2 in oxygen-dependent transactivation of mitochondrial proteins has also been proposed [64]. An isoform of *cytochrome c oxidase subunit 4* (*COX4I2*), which is exclusively expressed in the lung, trachea, and placenta at high levels and in the heart and brain at low levels, contains a putative oxygen responsive element (ORE) in its promoter region of the nucleic genome. The ORE is also found in the promoter of *CHCHD2*. CHCHD2 binds to ORE and transcriptionally upregulates both *COX4I2* and *CHCHD2* itself in cooperation with Notch-associated transcription factor RBP-Jk/CSL/CBF1 under conditions of low oxygen, while wild-type CHCHD10, but not pathogenic variants, acts as a negative regulator of ORE-mediated translation [64,65]. Moreover, CHCHD2 along with CHCHD10 interacts with complex IV subunits, leading to increased oxygen consumption and reduced ROS production [66]. In the regulation of complex IV, the phosphorylation of CHCHD2 at Y99 by Abl2 kinase seems to be involved [67]. However, the direct interaction between complex IV and CHCHD2 failed to be detected in our study and Y99 residue is not conserved in lower organisms such as *Drosophila* and yeast [50]. For the roles of CHCHD10 in the nucleus, opposite results were reported in another study, where both wild-type CHCHD10 and ALS-FTD-associated mutants activated nucleus-encoded mitochondrial complex I genes, *NADH dehydrogenase iron-sulfur protein 3* and *NADH dehydrogenase-1ß subcomplex subunit 6* [52]. Thus, further studies in terms of the roles of CHCHD2 and CHCHD10 in the nucleus would be warranted.

The expression pattern of CHCHD2 and CHCHD10 proteins appears to be similar at tissue levels and relatively high in the heart, liver, pancreas, and skeletal muscle while low in the forebrain and spinal cord in mice [51]. The immunohistochemical analysis has revealed that CHCHD2 and CHCHD10 immunosignals are rich in dopaminergic neurons of the substantia nigra, pyramidal neurons of the cortex and the hippocampus, and motor neurons in the anterior horn of the spinal cord. These are affected in PD, AD, and ALS–FTD [51,57]. Another study reported that the immunoreactivity of CHCHD2 is higher than that of CHCHD10 in dopaminergic neurons in the midbrain [58].

## 8. Binding Partners of CHCHD2 and CHCHD10

**Figure 4:**
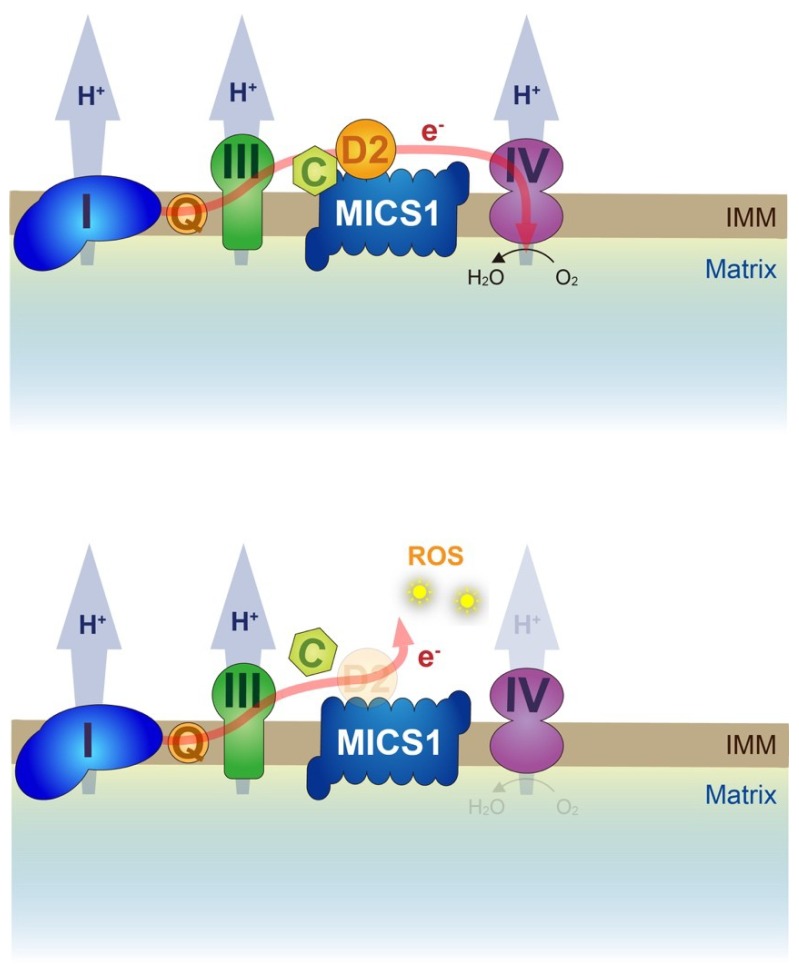
Working hypothesis for a role of CHCHD2 in the respiratory complex. (**Upper**) CHCHD2 (D2) along with MICS1 retains cytochrome c (c) in the respiratory complex, which stabilize electron flow from complex III to complex IV. (**Lower**) Loss of CHCHD2 destabilizes cytochrome c, leading to electron leak and subsequent ROS generation. IMM, inner mitochondrial membrane.

In human cultured cells, CHCHD2 has been suggested to be involved in apoptosis pathways through direct interaction with BCL-xL, an anti-apoptotic BCL-2-related protein, to prevent BAX oligomerization and subsequent cytochrome c release from the IMS [68]. A CHCHD2-binding protein, MICS1, which was originally characterized as a cytochrome c-binding anti-apoptotic protein, suppressing the cytochrome c release, regulates cristae organization on the IMS in HeLa cells [69]. MICS1 overexpression suppresses the defects of cristae organization by loss of *CG5010* in *Drosophila*, indicating that CHCHD2 in concert with MICS1 stabilizes cytochrome c in the respiratory complex, which would improve the electron transfer from complex III to complex IV (Figure 4) [50].

CHCHD2 and CHCHD10 form both homodimer and heterodimer, although the physiological meanings remain unclear [50,51,56,58]. Some pathogenic mutations may affect the dimerization and/or mitochondrial localization, although the observations were different in each study. For instance, CHCHD10 R15L and S59L do not affect dimerization but may rather disturb normal mitochondrial localization [51] while CHCHD10 R15L is normally localized at the mitochondria, as shown in another study [56]. A thorough study using *CHCHD2* and/or *CHCHD10* knockout cultured cells revealed that CHCHD2 promotes CHCH10 oligomerization [57]. Endogenous CHCHD10 is present as a monomer in the absence of CHCHD2, while endogenous CHCHD2 forms a homodimer and promotes the formation of the CHCHD2/CHCHD10 heterodimer. CHCHD2 is post-translationally stabilized upon the reduction of mitochondrial membrane potential (∆ψm) but not the changes in electron flux or ROS generation, facilitating CHCHD2/CHCHD10 heterodimer formation [57,70,71]. The regulation mechanism of their protein levels remains to be resolved, but the involvement of ∆ψm-dependent mitochondrial proteases and/or the proteasome is expected [57,72]. In *Drosophila*, CG5010 also responds to mitochondrial DNA damage and mitochondrial unfolded protein stress, and is stabilized on the PD-associated *PINK1*, *Parkin*, or *DJ-1*-defefient genetic backgrounds [50]. Thus, CHCHD2 and CHCHD10 are suggested to have a role in relieving mitochondrial stress, in addition to the roles in OXPHOS regulation.

**Figure 5:**
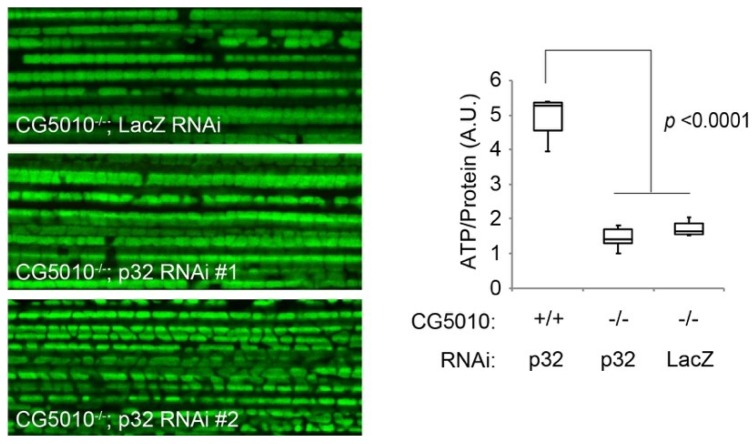
No evidence of genetic interaction between *Drosophila CHCHD2/D10* orthologue, *CG5010,* and *p32*. (**Left**) Mitochondria in the indirect flight muscles were visualized by mitoGFP (green). Although *p32*-deficient flies are lethal, muscle-specific reduction of *p32* does not affect muscular mitochondria. Reduction of *p32* in *CG5010* null flies does not change the mitochondrial morphology. Two independent *p32* RNAi lines obtained from the NIG-fly were employed. *LacZ* RNAi served as mock control. (**Right**) Loss of *CG5010* resulted in reduced ATP production (*p* < 0.0001, *CG5010^+/+^* with *p32* RNAi *vs*. *G5010^−/−^* with *p32* RNAi by Tukey–Kramer’s test), which was not affected by *p32* inactivation (*p* = 0.5546, *G5010^−/−^* with *p32* RNAi *vs*. *G5010^−/−^* with *LacZ* RNAi). *n* = 5–6 in each group. A.U., arbitrary units. Transgenes were driven by muscle-specific *MHC-GAL4* driver.

Both CHCHD2 and CHCHD10 also associate with a mitochondrial matrix protein p32/HABP1/C1qBP/gC1qR, although the binding mode of IMS-resident CHCHD2 and CHCHD10 with p32 remains unclear [51,56,73]. The loss of *p32* causes developmental defects in mouse embryos with severe dysfunction of the mitochondrial respiratory chain due to impaired mitochondrial protein synthesis, suggesting that p32 is involved in mitochondrial translation mediated by mitoribosomes [74]. The role of p32 in neurotransmitter release at synapses was reported in *Drosophila*, suggesting that p32 regulates the Ca^2+^-buffering ability of mitochondria [75]. Although p32 is suggested to be involved in a variety of critical mitochondrial functions, our study using *Drosophila* failed to detect significant genetic interaction between *p32* and *CG5010*, in spite of the conserved physical association (Figure 5).

CHCHD2 and CHCHD10 appear to reside in a high molecular weight complex of approximately 220 kDa [56]. In a glucose-free medium containing galactose, cells are forced to rely predominantly on OXPHOS rather than glycolysis for ATP production. Under this condition, CHCHD2 and CHCHD10 are upregulated and exist in a new complex of approximately 40 kDa, which indicates the rearrangement of CHCHD2- and CHCHD10-containing complexes in response to OXPHOS activity [56].

## 9. Pathogenesis caused by Twin CHCH Proteins

Mitochondrial defects have long been implicated in the etiology of PD as genetic evidence. Early-onset PD-causative genes, *PINK1* and *Parkin*, are well recognized to regulate mitochondrial quality control, which includes the arrest of mitochondrial transport and removal of damaged mitochondria through mitophagy [76]. Interestingly, the immune response and autophagy-associated *TBK1*, *Optineurin*, *VCP/p97*, and *SQSTM1/p62*, which are causative genes of ALS–FTD, have been shown to be involved in PINK1-Parkin-mediated mitophagy [77,78,79]. Another early-onset PD-causative gene, *DJ-1*, demonstrates resistance to oxidative stress [80,81].

Although *CG5010* is upregulated in *PINK1-*, *Parkin-*, and *DJ-1*-deficient flies, probably due to chronic reduction of ∆ψm, there was no evidence that CHCHD2/CHCHD10 and PINK1-Parkin signaling or DJ-1 directly interact [50]. Rather PINK1-Parkin activation exacerbated the *CG5010* mitochondrial phenotypes, suggesting that PINK1-Parkin signaling tried to remove all of the defected mitochondria by *CG5010* loss. A similar conclusion was reported by another study using human cultured cells [57].

TDP-43 is a heterogeneous ribonucleoprotein that plays a major role in regulating RNA splicing, stability, and transport. In ALS and FTD patients, the accumulation of TDP-43 in cell bodies of neurons in affected regions is often observed, indicating that a unifying pathology underlies the ALS–FTD spectrum. Mitochondrial mislocalization of TDP-43 and its involvement in neurotoxicity of ALS-FTD have also been reported [82]. Loss of CHCHD10 or ALS–FTD-associated CHCHD10 R15L and S59L mutants promotes cytosolic and mitochondrial localization of TDP-43 [52]. Another study comparing the effects of benign Jokela type spinal muscular atrophy (SMAJ)-linked CHCHD10 G66V and the more severe ALS–FTD-associated CHCHD10 S59L showed that the accumulation of phosphorylated TDP-43 is comparable among these mutants. Rather, MICOS assembly defects and mitochondrial fragmentation are well correlated with the severity of the disease [55]. Thus, the molecular relationship between CHCHD10 mutations and TDP-43 accumulation would need further study. 

**Figure 6:**
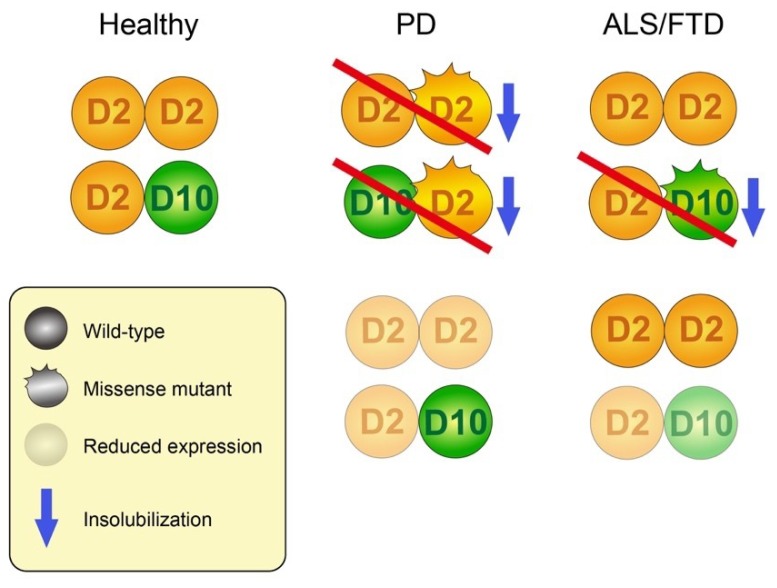
Working hypothesis showing that CHCHD2 and CHCHD10 produce a complicated disease spectrum. CHCHD2 forms a homodimer and stimulates heterodimerization with CHCHD10, which may be important to maintain mitochondrial activity [50,57]. In PD, CHCHD2 T61I mutation facilitates its insolubilization, which should involve the partners [50,57]. Splice-site mutation (c.300 + 5G > A) of *CHCHD2* and variants in the untranslated region may reduce CHCHD2 expression [7,32]. In both cases, functional CHCHD2 is reduced. In ALS–FTD, CHCHD10 G59L and G66V mutations facilitate their insolubilization, which should involve wild-type CHCHD2 [57]. Variants in the untranslated region of *CHCHD10* or truncated CHCHD10 mutants that cannot bind to CHCHD2 may also cause pathogenesis due to haploinsufficiency, although there are no experimental data so far. In both cases, the CHCHD2 homodimer will be intact.

Although *CHCHD2*- and *CHCHD10*-linked neurodegenerative diseases are dominantly inherited, the findings of their mutations leading to premature protein truncation suggest mitochondrial phenotypes arise from haploinsufficiency. Supporting this idea, the expression of PD-associated human CHCHD2 mutants on a wild-type genetic background, but not *CG5010* null background, does not produce obvious mitochondrial phenotypes in *Drosophila* [50]. However, another *Drosophila* study showed that the overexpression of human CHCHD2 mutants on a wild-type genetic background leads to mitochondrial degeneration and dopaminergic neuron loss. This may be attributed to higher levels of CHCHD2 ectopic expression and Myc-tag addition to CHCHD2, as even wild-type CHCHD2 expression caused a reduced electrophysiological response and shorter lifespan compared with a no transgene control. However, the finding that CHCHD2 T61I, which can form dimers with CHCHD2 [50] or CHCHD10 [58,83], tends to be insoluble suggests that at least CHCHD2 T61I has an aspect of toxic gain-of-function [50,57]. Similarly, a tendency of CHCHD10 S59L and G66V for insolubilization was shown [57]. Thus, the property of twin CHCH proteins to form homo- and heterodimer and the consequence of mutations in terms of solubility and dimer formation would produce a wide range of clinical and pathological manifestations (Figure 6).

## 10. Conclusions and Perspective

Although the mutation frequency of *CHCHD2* and *CHCHD10* is relatively low, the fact that many pathogenic and risk variants of the two genes have been isolated from patients with a variety of neurodegenerative diseases strongly suggests that the dysfunctions of these twin proteins underlie the pathogenesis of major neurodegenerative diseases. Considering the observations that CHCHD2 and CHCHD10 form different patterns of mutant complex, there could be multiple modes of pathogenesis in these diseases. In addition, not a few mutations of both genes are found in sporadic neurodegenerative diseases, raising the possibility that the two genes are key factors in polygenic hereditary diseases. Thus, it would be important to examine both gene mutations simultaneously. Finally, what mechanism by the dysfunctions of the twin proteins determines the affected nervous system? The issue is the “twin paradox” in current neurology.

## Abbreviations

AD	Alzheimer’s disease
ALS	Amyotrophic lateral sclerosis
CHCH	Coiled-coil-helix-coiled-coil-helix
CMT2	Charcot–Marie–Tooth disease type 2
COX	Cytochrome c oxidase
DLB	Dementia with Lewy bodies
FTD	Frontotemporal lobe dementia
IMS	Mitochondrial intermembrane space
Mia40	Mitochondrial intermembrane space import and assembly protein 40 kDa
MTS	Mitochondrial targeting sequence
MICOS	Mitochondrial contact site complex
OXPHOS	Oxidative phosphorylation
PD	Parkinson’s disease
ROS	Redox oxygen species
SMAJ	Jokela type spinal muscular atrophy
TDP-43	TAR DNA binding protein of 43 kDa
∆ψm	Mitochondrial membrane potential
